# Comparative genomic and phenotypic analysis of *Corynebacterium macginleyi* from ocular infections

**DOI:** 10.1128/msphere.00408-26

**Published:** 2026-08-04

**Authors:** Qiheng Yuan, Yangyang Shen, Xiaowei Liu, Bianjin Sun, Meiqin Zheng

**Affiliations:** 1 National Clinical Research Center for Ocular Diseases, Eye Hospital, Wenzhou Medical University 224181, Wenzhou, China; 2 Wenzhou Key Laboratory of Sanitary Microbiology, Key Laboratory of Laboratory Medicine, Ministry of Education, School of Laboratory Medicine and Life Science, Wenzhou Medical University 26453, Wenzhou, Zhejiang, China; Tokai Daigaku Igakubu Daigakuin Igaku Kenkyuka, Isehara, Kanagawa, Japan

**Keywords:** *Corynebacterium macginleyi*, ocular infections, genomic analysis, phenotypic analysis

## Abstract

*Corynebacterium macginleyi* is an opportunistic pathogen linked to ocular infections, but comprehensive genomic studies remain scarce, especially in China. This study characterized its genomic features, virulence potential, and antimicrobial resistance using 13 clinical isolates and 27 public genomes. Methods included matrix-assisted laser desorption/ionization time-of-flight mass spectrometry (MALDI-TOF MS), average nucleotide identity (ANI), whole-genome sequencing, Clusters of Orthologous Groups/Kyoto Encyclopedia of Genes and Genomes annotation, core-single nucleotide polymorphism phylogeny, virulence and resistance gene analysis, and Clinical and Laboratory Standards Institute susceptibility testing. All isolates were accurately identified as *C. macginleyi*, with MALDI-TOF MS and ANI outperforming conventional methods. Phylogenetic analysis revealed three clades with geographic clustering. Functional profiles were centered on metabolism. Virulence genes were conserved, mainly mediating adhesion, stress response, and immune evasion. Resistance genes were limited but diverse, dominated by *erm(X*). Phenotypic testing showed frequent macrolide resistance, while most isolates remained susceptible to the other tested antimicrobials; fluoroquinolone resistance was detected in a subset of isolates. Isolate B64 exhibited a unique phylogenetic position, higher virulence, and resistance gene burden, consistent with multidrug resistance. In conclusion, *C. macginleyi* shows geographic diversity, conserved virulence, and evolving resistance, supporting precise identification and continuous surveillance.

**IMPORTANCE:**

The precise molecular mechanisms underlying the pathogenicity of *Corynebacterium macginleyi* are not fully elucidated, and large-scale genomic and epidemiological studies are still limited, particularly in certain regions such as China. Therefore, comprehensive investigations integrating whole-genome sequencing with clinical data are urgently needed to better understand its genetic characteristics, virulence-associated traits, and antimicrobial resistance profiles. Such efforts will provide a critical foundation for improving diagnostic accuracy and optimizing therapeutic strategies for ocular infections caused by this organism.

## INTRODUCTION

*Corynebacterium macginleyi* is increasingly recognized as an opportunistic pathogen associated with ocular surface infections, particularly bacterial keratitis and conjunctivitis. Since its first isolation and description by Riegel et al. in 1995 (1), accumulating clinical evidence has highlighted its relevance in ophthalmic infections. Notably, Joussen et al. identified *C. macginleyi* as a conjunctiva-specific pathogen, emphasizing its strong association with ocular surface disease (2). Subsequent epidemiological investigations have confirmed its presence among the predominant bacterial species isolated from conjunctivitis and keratitis cases, although its reported prevalence varies across geographic regions and study populations (3, 4). More recent large-scale clinical studies and microbiome-based analyses further support its increasing detection frequency in ocular infections, with some reports indicating that *C. macginleyi* ranks among the leading *Corynebacterium* species isolated from the conjunctival sac and keratitis samples (5–8). These findings collectively indicate that *C. macginleyi* represents a clinically significant but often underrecognized pathogen in ocular infections.

With regard to pathogenicity, *C. macginleyi* is generally considered to possess relatively limited classical virulence factors compared with highly invasive ocular pathogens; however, it exhibits several adaptive features that may contribute to infection. These include the production of enzymes such as lipases and proteases, as well as metabolic adaptations that facilitate survival on the lipid-rich ocular surface (9–11). In addition, interactions with host immune responses and persistence within the conjunctival microenvironment may promote chronic or recurrent infections (9). Emerging evidence from ocular microbiome studies suggests that *C. macginleyi* may act as a commensal organism under physiological conditions but shift toward pathogenicity under conditions of dysbiosis or ocular surface disruption (12, 13).

Advances in microbiological diagnostics, particularly the application of MALDI-TOF MS and whole-genome sequencing, have significantly improved the identification and characterization of *C. macginleyi* isolates (14). These approaches have provided new insights into its genomic structure, resistance mechanisms, and phylogenetic relationships. Importantly, emerging antimicrobial resistance—especially to fluoroquinolones—has been increasingly reported, raising concerns regarding empirical treatment strategies for ocular infections (15, 16). Recent studies have further highlighted the presence of resistance-associated mutations and the growing clinical challenge posed by multidrug-resistant isolates (15–17).

Despite these advances, several knowledge gaps remain. The precise molecular mechanisms underlying the pathogenicity of *C. macginleyi* are not fully elucidated, and large-scale genomic and epidemiological studies are still limited, particularly in certain regions such as China (5). Therefore, comprehensive investigations integrating whole-genome sequencing with clinical data are urgently needed to better understand its genetic characteristics, virulence-associated traits, and antimicrobial resistance profiles. Such efforts will provide a critical foundation for improving diagnostic accuracy and optimizing therapeutic strategies for ocular infections caused by this organism.

## MATERIALS AND METHODS

### Sample collection

A total of 13 *C. macginleyi* isolates were collected from infection sites of patients with conjunctivitis or keratitis at the Eye Hospital of Wenzhou Medical University. In addition, 27 publicly available whole-genome sequences of *C. macginleyi* were downloaded from the NCBI database, with detailed information provided in Table S1.

### Bacterial identification and DNA extraction

Bacterial colonies were inoculated into 5 mL of brain heart infusion broth supplemented with 5% fetal bovine serum and incubated at 37°C with shaking at 150 rpm for enrichment. After 3–4 days of incubation, the isolates were subjected to microscopic examination, biochemical identification using the API Coryne system (analytical profile index Coryne system), and MALDI-TOF mass spectrometry analysis (MALDI-TOF MS). MALDI-TOF MS identification was performed using the MS600 microbial mass spectrometry system (Autobio Diagnostics, Zhengzhou, China) with the manufacturer’s proprietary microbial protein fingerprint database, according to the manufacturer’s instructions. The API Coryne test strip, a biochemical identification system for coryneform bacteria, was used according to the manufacturer’s instructions (bioMérieux, Marcy-l’Étoile, France). Genomic DNA of *C. macginleyi* was extracted using the GK1072 bacterial genomic DNA extraction kit (Jierui, China) according to the manufacturer’s instructions.

### Whole-genome sequencing, quality control, assembly, and ANI analysis

Genomic DNA was extracted and subjected to next-generation sequencing on the Illumina platform (Berry Genomics, Beijing, China). Sequencing libraries were prepared following standard protocols, including DNA fragmentation, library construction, and sequencing reactions. The integrity of sequencing data was verified using MD5 checksums prior to downstream analysis.

Raw sequencing reads were processed using fastp for quality control, including filtering of low-quality reads, trimming of adapter sequences, and removal of reads containing excessive ambiguous bases (18). The resulting high-quality clean reads were then used for *de novo* genome assembly with SPAdes (v3.15.4) (19). Assembly quality was subsequently evaluated based on standard metrics.

To further determine the genomic similarity and confirm species identity, average nucleotide identity (ANI) analysis was performed on the assembled genome sequences. The ANI values were calculated by comparison with the reference genome (GCA_016889465.1). An ANI threshold of ≥95% was used as the criterion for species-level identification (20).

Unless otherwise specified, all software tools were executed using default parameters.

### Gene structure and functional annotation

Open reading frame sequences were annotated using Prokka v1.12 (21). Subsequently, protein sequences predicted by Prokka were functionally annotated using eggNOG-mapper (v2.0) (https://github.com/eggnogdb/eggnog-mapper) (22). Orthologous group assignment and functional annotation were performed based on the eggNOG database. To further characterize gene functions, the annotated sequences were mapped to the Clusters of Orthologous Groups (COG) and Kyoto Encyclopedia of Genes and Genomes (KEGG) databases.

### Phylogenetic analysis

Single nucleotide polymorphisms (SNPs) were identified from the Prokka-annotated data using Snippy (https://github.com/tseemann/snippy). Recombinant regions were subsequently removed using Gubbins (23). A phylogenetic tree was then constructed using IQ-TREE 3, and the resulting tree was visualized using ChiPlot (24).

### Virulence and antimicrobial resistance analysis

Virulence factors of *C. macginleyi* were annotated using the Virulence Factor Database (VFDB) database via the online platform (https://www.mgc.ac.cn) (25). Antimicrobial resistance genes were identified using AMRFinderPlus v4.0.22 (database version 2025-03-25.1; parameters: amrfinder –report_all_equal –plus –ident_min 0.95 –coverage_min 90) (26). Data visualization was performed using R software (v4.4.3).

### Antimicrobial susceptibility testing

Antimicrobial susceptibility testing was performed using the broth microdilution method in accordance with the Clinical and Laboratory Standards Institute (CLSI) guidelines for *Corynebacterium* spp*.* (27) and related coryneform bacteria. Fresh bacterial colonies were suspended to a turbidity equivalent to a 0.5 McFarland standard and inoculated into broth microdilution panels containing cation-adjusted Mueller–Hinton broth supplemented with lysed horse blood (CAMHB + LHB). The tested antimicrobial agents included erythromycin, clindamycin, gentamicin, vancomycin, trimethoprim-sulfamethoxazole (TMP-SMX), tetracycline, doxycycline, linezolid, penicillin, ciprofloxacin, and levofloxacin. The panels were incubated at 35°C under ambient air conditions for 24–48 h before MIC determination. MICs were interpreted according to CLSI criteria when applicable (28).

## RESULTS

### Clinical characteristics of the isolates

Clinical diagnoses or background information corresponding to each isolate are summarized in Table 1. Most isolates were obtained from patients diagnosed with keratitis. The cohort comprised eight male and five female patients, indicating a slight male predominance. The age of patients ranged from 1 month to 75 years, with a median age of 52 years. Notably, two cases occurred in infants (1 month old), while the remaining patients were predominantly adults. Overall, the age distribution suggested that *C. macginleyi* infection can occur across a wide age range, with a higher proportion observed in middle-aged and elderly individuals.

**TABLE 1:** Clinical strain information^*a*^

Strain	Sex	Age (years)	Disease
B18	Male	25	Keratitis and post-operative care for lacerations
B27	Male	1 month	Keratitis
B29	Female	43	Keratitis
B32	Male	67	Keratitis
B33	Female	65	Keratitis
B40	Female	62	Corneal thermal burn
B64	Male	62	Keratitis and post-deep lamellar keratoplasty
B66	Male	52	Keratitis
B77	Female	1 month	Keratitis
B78	Male	46	Keratitis
B83	Male	65	Corneal thermal burn
B84	Male	52	Keratitis
B88	Female	75	Keratitis

^*a*^ All isolates were obtained from corneal scrapings.

### Culture characteristics and mass spectrometry identification results

**Fig 1:**
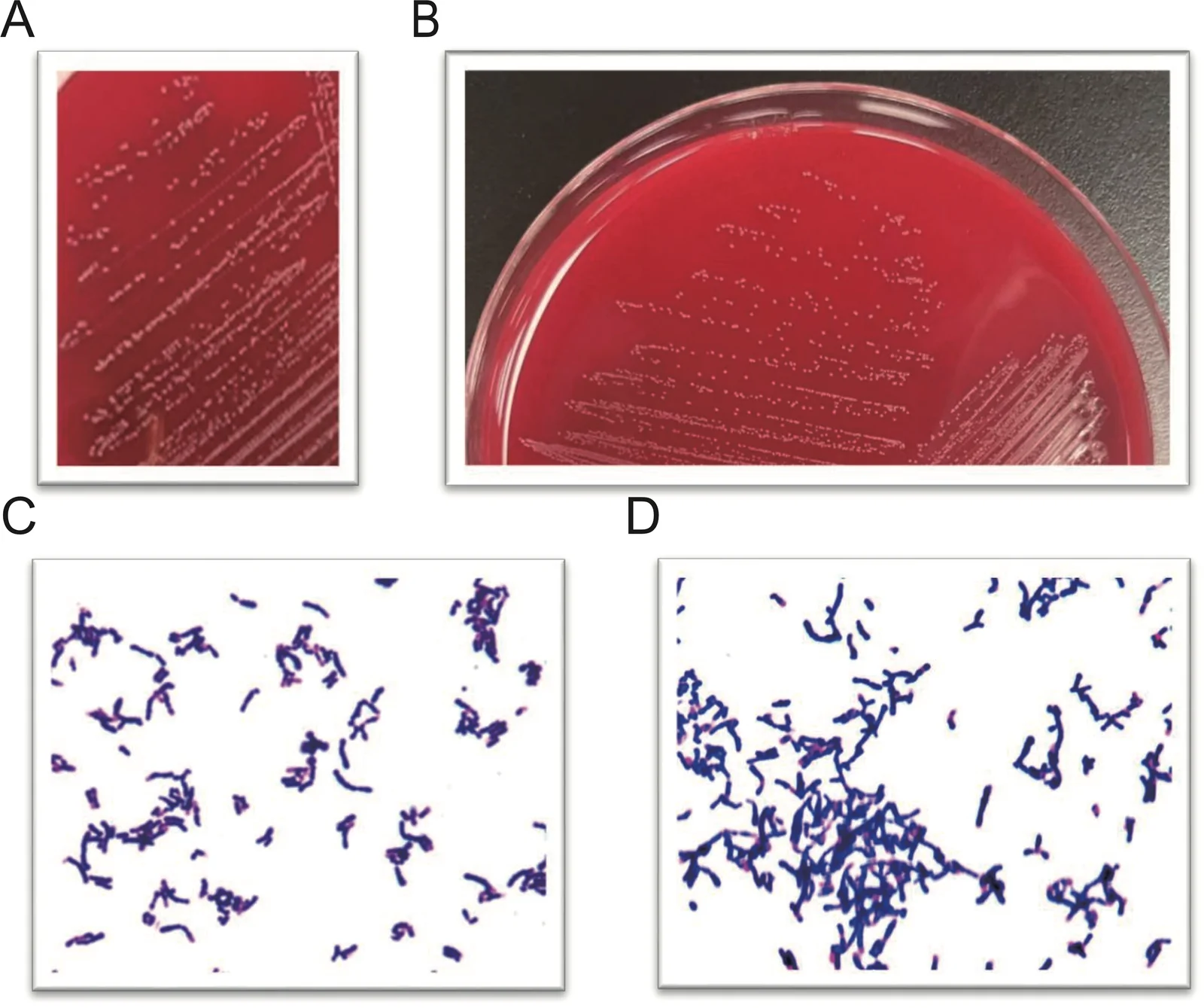
Culture characteristics and Gram staining of *Corynebacterium macginleyi*. (**A and B**) Colony morphology of *Corynebacterium macginleyi* cultured on blood agar plates. (**C and D**) Microscopic images of Gram-stained *Corynebacterium macginleyi*.

*C. macginleyi* exhibited slow growth on standard blood agar plates. After 3–5 days of incubation, small colonies approximately 0.5 mm in diameter were observed, appearing gray-white, round, and translucent (Fig. 1A and B). Neither 5% CO_2_ nor anaerobic conditions promoted the growth or proliferation of *C. macginleyi*. Gram staining revealed coryneform gram-positive rods with palisading arrangement and club-shaped morphology (Fig. 1C and D).

As shown in Table 2, biochemical identification using the API Coryne system showed that 3 of the 13 isolates were identified only at the genus level as *Corynebacterium* spp*.*, whereas the remaining 10 isolates were identified as *Corynebacterium propinquum*. In contrast, both MALDI-TOF mass spectrometry and ANI analysis consistently identified all isolates as *C. macginleyi*, which was inconsistent with the API identification results.

**TABLE 2:** Bacterial identification results by different methods^*a*^

Strain	API Coryne test strips	Mass spectrometry identification (score range 8.0–9.6)	Reference strain *Corynebacterium macginleyi* (GCA_016889465.1)	
			ANIb values (%)	ANIm values (%)
B18	*Corynebacterium propinquum*	*Corynebacterium macginleyi*	98.55	98.65
B27	*Corynebacterium propinquum*	*Corynebacterium macginleyi*	98.61	98.69
B29	*Corynebacterium propinquum*	*Corynebacterium macginleyi*	98.5	98.64
B32	*Corynebacterium*	*Corynebacterium macginleyi*	98.49	98.61
B33	*Corynebacterium propinquum*	*Corynebacterium macginleyi*	98.42	98.54
B40	*Corynebacterium*	*Corynebacterium macginleyi*	98.34	98.51
B64	*Corynebacterium propinquum*	*Corynebacterium macginleyi*	95.75	95.94
B66	*Corynebacterium propinquum*	*Corynebacterium macginleyi*	98.29	98.45
B77	*Corynebacterium propinquum*	*Corynebacterium macginleyi*	98.25	98.42
B78	*Corynebacterium propinquum*	*Corynebacterium macginleyi*	98.36	98.49
B83	*Corynebacterium propinquum*	*Corynebacterium macginleyi*	98.37	98.53
B84	*Corynebacterium propinquum*	*Corynebacterium macginleyi*	98.39	98.54
B88	*Corynebacterium*	*Corynebacterium macginleyi*	98.35	98.5
				

^*a*^ API, analytical profile index; ANI, average nucleotide identity.

### Functional annotation based on COG and KEGG databases

**Fig 2:**
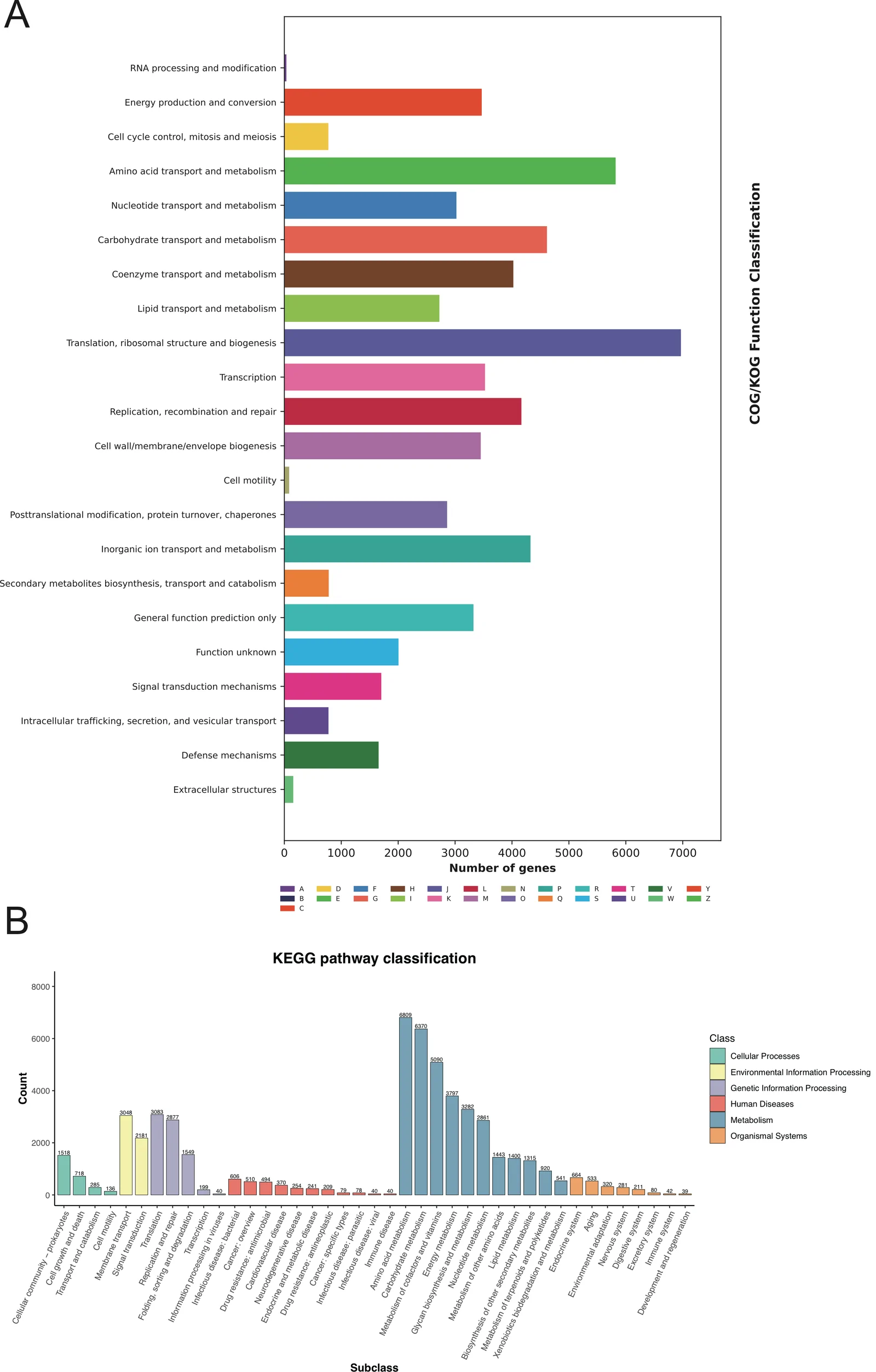
Functional annotation of *Corynebacterium macginleyi* based on COG and KEGG analyses. (**A**) COG functional classification, with different colors representing distinct functional classes. (**B**) KEGG pathway annotation, where different colors indicate functional classes and subclasses are labeled.

Functional annotation of genes from 40 *C. macginleyi* strains (13 isolates and 27 publicly available genomes) was performed using the COG and KEGG databases (Fig. 2).

COG classification (Fig. 2A) showed that annotated genes were predominantly enriched in translation, ribosomal structure, and biogenesis (J) and amino acid transport and metabolism (E). Functional categories related to carbohydrate transport and metabolism (G), replication, recombination, and repair (L), and inorganic ion transport and metabolism (P) were also well represented. In contrast, genes associated with cell motility (N), extracellular structures (W), and RNA processing and modification (A) were minimally represented.

Consistent with the COG results, KEGG pathway analysis (Fig. 2B) demonstrated that most genes were assigned to metabolic pathways, followed by genetic information processing and environmental information processing. At the subclass level, pathways involved in amino acid metabolism, carbohydrate metabolism, and metabolism of cofactors and vitamins were the most abundant. Pathways related to translation, replication, and repair, and protein folding and degradation were also highly represented, while those associated with cell motility and organismal systems were relatively scarce. Overall, genes assigned to metabolism-related categories were the most abundant in both COG and KEGG annotations, whereas genes associated with cell motility and extracellular structures were relatively uncommon.

### Phylogenetic analysis

**Fig 3:**
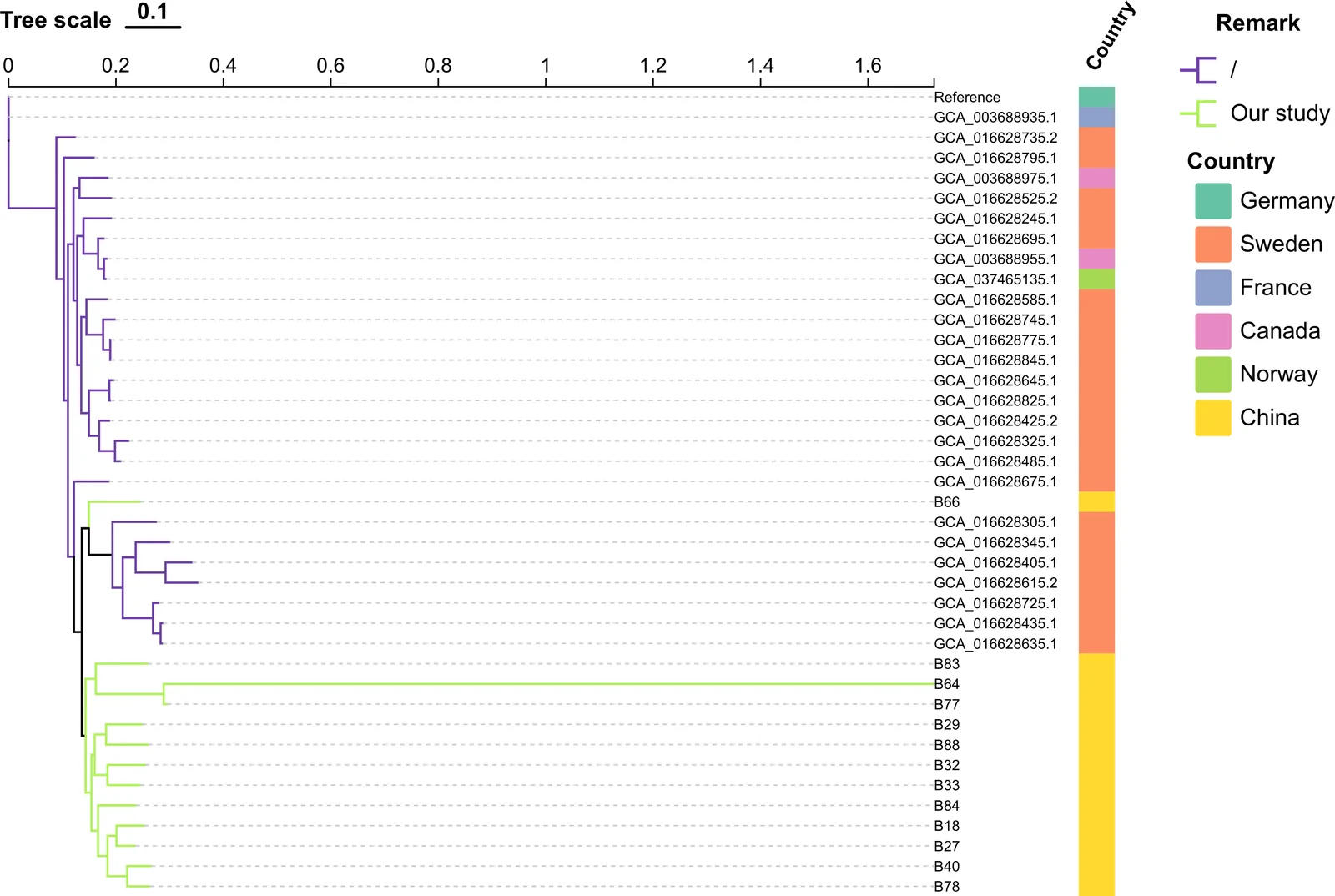
Phylogenetic tree of *Corynebacterium macginleyi*. Branches distinguish strains derived from this study and those from public databases. The accompanying bar plot indicates the country of origin for each strain.

Phylogenetic analysis based on core-genome SNPs was performed for 40 *C. macginleyi* genomes, including 13 clinical isolates from this study and 27 publicly available genomes (Fig. 3). The resulting phylogeny separated the strains into three major clades. The first major clade mainly comprised publicly available strains from Europe and Canada. The second clade included 12 isolates from China. The third branch was represented by strain B66 from this study, which clustered with seven publicly available strains from Sweden and was separated from the other two major clades.

### Virulence gene profiling based on VFDB

**Fig 4:**
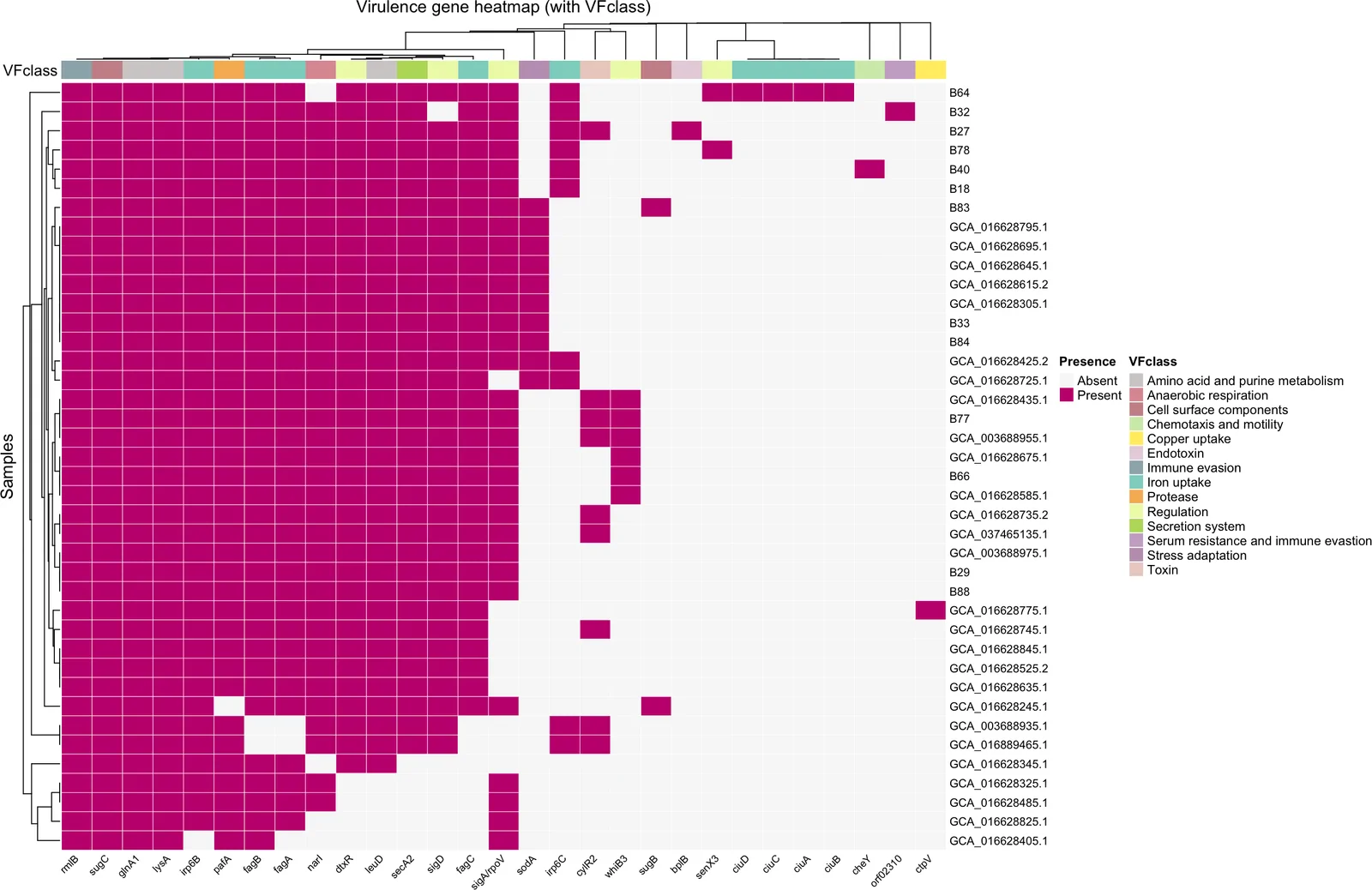
Virulence gene annotation of *Corynebacterium macginleyi*. Different colors represent distinct classes of virulence factors.

Virulence gene distribution among 40 *C. macginleyi* strains was analyzed using the VFDB database (Fig. 4). Overall, several virulence-associated genes were detected in most strains. Genes involved in amino acid and purine metabolism, such as *glnA1* and *lysA*, were widely distributed. Iron uptake-related genes, including *irp6B* and *fagA*, *fagB*, and *fagC*, were also broadly present among the strains. The *rmlB* gene, annotated as being associated with immune evasion, was detected in most strains. Several virulence-associated genes showed uneven distribution among strains. For example, strain B64 harbored the *ciuA*, *ciuB*, *ciuC*, and *ciuD* iron uptake-related genes. Cluster analysis showed that most isolates shared similar virulence-associated gene profiles, whereas a few strains exhibited distinct gene presence or absence patterns.

### Antibiotic resistance gene profiling

**Fig 5:**
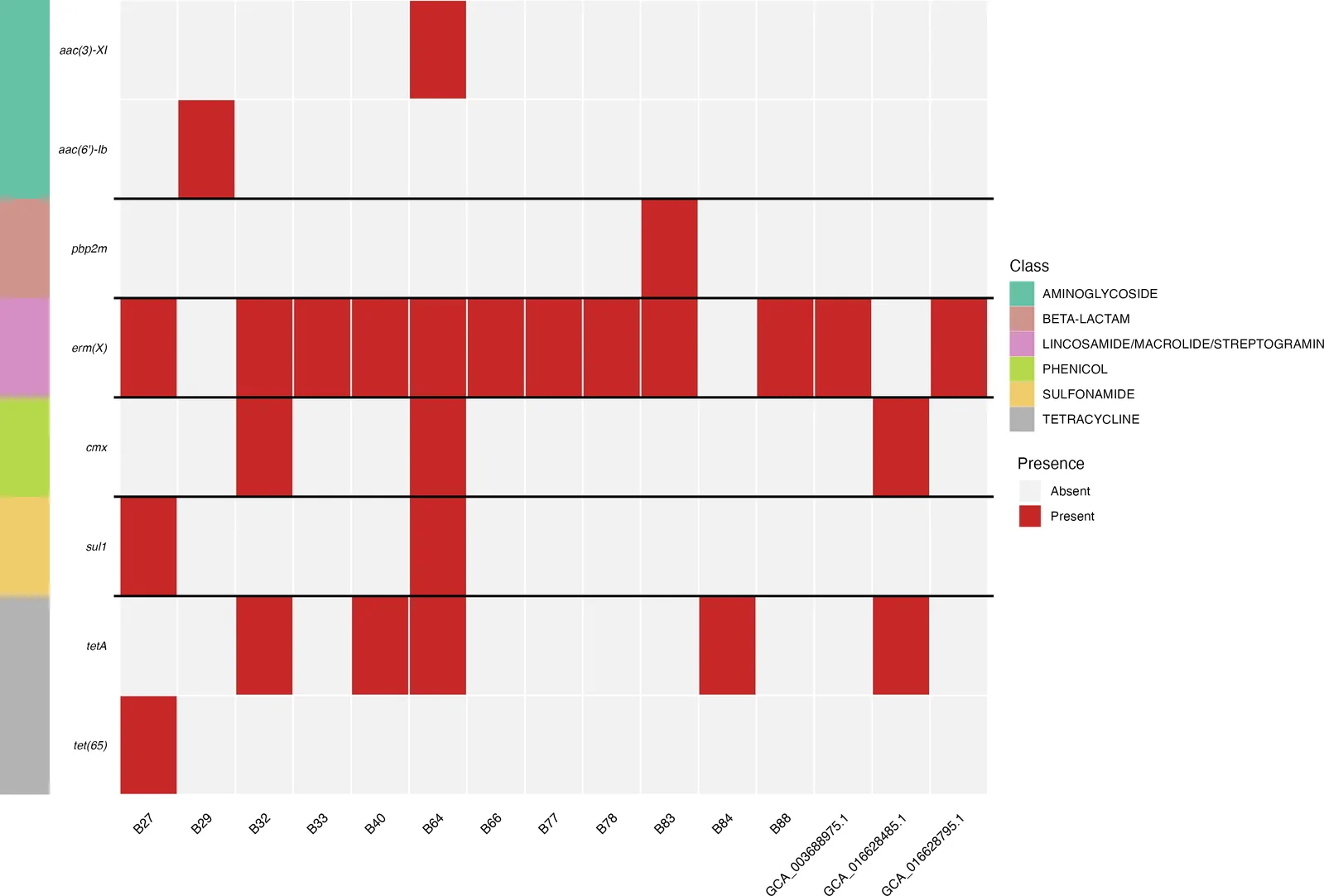
Antibiotic resistance gene annotation of *Corynebacterium macginleyi*. Different colors represent distinct classes of antibiotic resistance genes.

Antibiotic resistance genes identified in *C. macginleyi* strains are shown in Fig. 5. Overall, resistance genes were unevenly distributed among the strains, with a limited number of gene types detected. Among them, *erm(X)*, associated with macrolide–lincosamide–streptogramin resistance, was the most prevalent and widely distributed gene across the strains. Genes related to tetracycline resistance [e.g., *tetA* and *tet(65)*] and phenicol resistance (*cmx*) were detected in several strains but showed a sporadic distribution pattern. In contrast, genes conferring resistance to aminoglycosides [e.g., *aac(3)-XI* and *aac(6′)-Ib*], β-lactams (*pbp2m*), and sulfonamides (*sul1*) were rarely observed and limited to a few isolates.

The strains from this study carried a greater number and diversity of annotated resistance genes than the publicly available strains from Europe and other regions included in this analysis. In particular, strain B64 harbored five resistance genes, including *aac(3)-XI*, *erm(X)*, and *cmx*, representing the highest resistance-gene count among the analyzed isolates.

### Antimicrobial susceptibility testing

Antimicrobial susceptibility testing showed varied resistance patterns among the 13 *C. macginleyi* isolates (Table 3). All 13 isolates were resistant to erythromycin. For clindamycin, nine isolates were resistant, two showed intermediate susceptibility, and two were susceptible.

**TABLE 3:** Antimicrobial susceptibility testing results^*a*^

Strain	Erythromycin	Clindamycin	Gentamicin	Levofloxacin	Vancomycin	TMP-SMX	Tetracycline	Doxycycline	Linezolid	Penicillin	Ciprofloxacin
B18	R	S	S	S	S	S	S	S	S	S	S
B27	R	R	S	S	S	R	R	S	S	S	S
B29	R	I	S	S	S	S	S	S	S	S	S
B32	R	R	S	S	S	R	R	S	S	S	S
B33	R	R	S	S	S	S	S	S	R	S	S
B40	R	R	I	S	S	S	S	S	S	R	S
B64	R	R	I	R	S	R	S	S	S	I	R
B66	R	R	S	S	S	S	S	S	S	S	S
B77	R	R	S	S	S	S	S	S	S	S	S
B78	R	R	S	S	S	S	S	S	S	S	S
B83	R	R	S	I	S	R	S	S	S	R	S
B84	R	I	S	R	S	S	S	S	S	S	R
B88	R	S	S	S	S	S	S	S	S	I	S

^*a*^ S, susceptible; I, intermediate; R, resistant; TMP-SMX, trimethoprim-sulfamethoxazole.

All isolates were susceptible to vancomycin and doxycycline. Most isolates were susceptible to gentamicin, levofloxacin, tetracycline, linezolid, penicillin, ciprofloxacin, and TMP-SMX, although resistance or intermediate susceptibility was observed for several agents. Tetracycline resistance was observed in two isolates, TMP-SMX resistance in four isolates, penicillin resistance in two isolates, and linezolid resistance in one isolate. For levofloxacin, two isolates were resistant and one showed intermediate susceptibility. For ciprofloxacin, two isolates were resistant. Gentamicin resistance was not observed, although two isolates showed intermediate susceptibility.

Susceptibility profiles differed among isolates. Strain B64 showed resistance to erythromycin, clindamycin, TMP-SMX, levofloxacin, and ciprofloxacin and showed intermediate susceptibility to gentamicin and penicillin.

## DISCUSSION

In this study, we performed a comprehensive genomic and phenotypic analysis of *Corynebacterium macginleyi* isolates from ocular infections, providing new insights into its epidemiology, pathogenic potential, and antimicrobial resistance characteristics. Notably, this work represents one of the few systematic investigations of *C. macginleyi* in China, thereby contributing valuable data to the limited existing literature (5, 29).

Consistent with previous reports, *C. macginleyi* was confirmed as an important opportunistic pathogen associated with ocular surface infections, particularly keratitis and conjunctivitis (2, 4, 9). The clinical data in our study showed that most isolates were derived from ocular infections, mainly keratitis. Several cases were associated with ocular surface abnormalities or relevant clinical backgrounds, such as corneal involvement or prior ocular surgery. These observations indicate that ocular surface disruption was present in part of the cohort. However, because this study included a small number of cases and lacked a comparison group, our data do not establish that *C. macginleyi* preferentially infects compromised ocular surfaces or shows tissue tropism. The lipophilic characteristics of *C. macginleyi* may facilitate colonization of the lipid-rich ocular surface, including environments associated with Meibomian and Zeis glands, but this hypothesis requires further validation (30).

Our study also highlights important limitations in routine bacterial identification methods. API-based systems failed to accurately identify *C. macginleyi* at the species level, frequently misclassifying isolates as *Corynebacterium propinquum*. In contrast, MALDI-TOF MS and whole-genome ANI analysis showed complete concordance and provided reliable species-level identification (11, 14). Therefore, when lipophilic coryneform gram-positive rods are recovered in significant or predominant amounts from ocular specimens, particularly in a compatible clinical context, definitive identification by MALDI-TOF MS or molecular methods should be considered, as conventional biochemical methods have limited reliability for this organism. In addition, the slow growth observed in this study refers to growth on routine, non-lipid-enriched blood agar; as a lipophilic species, *C. macginleyi* may grow more rapidly on lipid-enriched media such as Tween 80-supplemented medium (31).

Phylogenetic analysis revealed that *C. macginleyi* strains could be broadly divided into three distinct clades, with clear geographic clustering patterns. Isolates from China formed a relatively independent lineage, while strains from Europe and Canada grouped into separate clusters. Similar clustering patterns were also observed in virulence and resistance gene profiles, suggesting that the population structure of *C. macginleyi* may be influenced by geographic factors. These findings indicate potential regional evolution and adaptation, which may contribute to differences in pathogenicity and antimicrobial resistance among strains from different locations. However, because the number and geographic distribution of publicly available genomes remain limited, these findings should be interpreted cautiously and should not be taken as definitive evidence of regional evolution or adaptation.

Genomic analysis showed that *C. macginleyi* harbors a conserved set of virulence-associated genes, mainly related to amino acid and purine metabolism, iron acquisition, and immune evasion. Among these, *glnA1* and *lysA* were widely distributed across the strains, suggesting that metabolic adaptation may contribute to bacterial survival and persistence in the ocular surface environment (32). In addition, the broad distribution of iron uptake-related genes, including *irp6B* and *fagA–C*, indicates that efficient iron acquisition may represent an important strategy for *C. macginleyi* during host colonization, particularly under the iron-restricted conditions imposed by the host (9, 11). The consistent presence of *rmlB*, a gene associated with immune evasion, further suggests that this organism may possess mechanisms that facilitate persistence in host tissues (33).

With regard to antimicrobial resistance, both genomic and phenotypic analyses demonstrated a relatively limited but heterogeneous resistance profile. The *erm(X*) gene was the most frequently detected annotated resistance gene in this study. Its distribution paralleled the frequent erythromycin resistance and clindamycin non-susceptibility observed among the isolates. Because functional validation was not performed and directly relevant evidence in *C. macginleyi* remains limited, this finding should be interpreted as an observed genotype-phenotype association rather than direct mechanistic proof. In contrast, resistance genes associated with other antibiotic classes were infrequently detected, and most isolates remained susceptible to these agents. The sporadic presence of additional resistance genes, including tetracycline, phenicol, aminoglycoside, and β-lactam-associated determinants, further highlights the inter-strain variability of antimicrobial resistance in *C. macginleyi*. These findings suggest that although the overall resistance gene repertoire of *C. macginleyi* is relatively limited, ongoing surveillance remains necessary to monitor the emergence of multidrug-resistant or regionally enriched resistant isolates. The clinical significance of antimicrobial resistance in *C. macginleyi* should be interpreted cautiously in ocular infections. Topical antibiotics are the preferred treatment for most bacterial keratitis and can achieve local concentrations substantially higher than systemic drug levels; therefore, susceptibility interpretations based on systemic breakpoints may not directly predict the efficacy of topical ophthalmic therapy. Nevertheless, these data remain useful for surveillance of emerging resistance, particularly fluoroquinolone or multidrug resistance.

Among all isolates, strain B64 deserves particular attention. In the phylogenetic tree, B64 showed a markedly longer branch length than most other strains, indicating substantial genetic divergence and suggesting that it may represent a relatively distinct sublineage within *C. macginleyi*. This genomic distinctiveness was accompanied by a unique virulence and resistance profile. Compared with other isolates, B64 carried a greater number of virulence-associated genes, including the *ciuABCD* iron uptake gene cluster, which may enhance its capacity for nutrient acquisition and adaptation under host-associated conditions (34). Therefore, *C. macginleyi* appears to contain a conserved core set of virulence-associated genes, together with strain-specific variations that may contribute to differences in pathogenic potential. In addition, B64 harbored a relatively larger repertoire of antimicrobial resistance genes, including *aac(3)-XI*, *erm(X*), and *cmx*, indicating an elevated resistance potential (35). Importantly, antimicrobial susceptibility testing further supported this genomic prediction. Taken together, these findings suggest that B64 may represent a potentially high-virulence and high-resistance strain.

This study has several limitations. First, the sample size was relatively small, and all isolates were obtained from a single center. Second, although antimicrobial susceptibility testing was performed to complement the genomic analysis, the exploration of resistance mechanisms was still primarily based on genomic annotation, and the underlying molecular mechanisms require further investigation.

In conclusion, this study provides a comprehensive characterization of *C. macginleyi* from ocular infections, highlighting its geographic diversity, conserved yet adaptable virulence profile, and emerging antimicrobial resistance. These findings underscore the importance of accurate identification and continuous surveillance of this organism. Future studies integrating functional experiments, multicenter sampling, and clinical outcome data are warranted to further elucidate its pathogenic mechanisms and guide effective therapeutic strategies.

## Data Availability

The draft genome assemblies generated in this study have been deposited in NCBI GenBank under BioProject accession number PRJNA1490348. The publicly available genomes analyzed in this study are listed in Table S1.

## SUPPLEMENTAL MATERIAL

Table S1 msphere.00408-26-s0001.xlsx Basic information of *Corynebacterium mastitidis* from public databases.

ASM does not own the copyrights to Supplemental Material that may be linked to, or accessed through, an article. The authors have granted ASM a non-exclusive, world-wide license to publish the Supplemental Material files. Please contact the corresponding author directly for reuse.
